# Cardiovascular risk and testosterone – from subclinical atherosclerosis to lipoprotein function to heart failure

**DOI:** 10.1007/s11154-021-09628-2

**Published:** 2021-02-22

**Authors:** Baris Gencer, Marco Bonomi, Maria Pia Adorni, Cesare R. Sirtori, François Mach, Massimiliano Ruscica

**Affiliations:** 1 Cardiology Division, Geneva University Hospitals, Geneva, Switzerland; 2 Department of Medical Biotechnology and Translational Medicine, Università degli Studi di Milano, Milan, Italy; 3 Department of Endocrine and Metabolic Diseases & Lab. of Endocrine and Metabolic Research, IRCCS Istituto Auxologico Italiano, Milan, Italy; 4 Department of Medicine and Surgery-Unit of Neurosciences, University of Parma, Parma, Italy; 5 Department of Pharmacological and Biomolecular Sciences, Università degli Studi di Milano, Milan, Italy

**Keywords:** Atherosclerosis, Hypogonadism, Cardiovascular risk, Cholesterol-efflux capacity, Inflammation, Testosterone

## Abstract

The cardiovascular (CV) benefit and safety of treating low testosterone conditions is a matter of debate. Although testosterone deficiency has been linked to a rise in major adverse CV events, most of the studies on testosterone replacement therapy were not designed to assess CV risk and thus excluded men with advanced heart failure or recent history of myocardial infarction or stroke. Besides considering observational, interventional and prospective studies, this review article evaluates the impact of testosterone on atherosclerosis process, including lipoprotein functionality, progression of carotid intima media thickness, inflammation, coagulation and thromboembolism, quantification of plaque volume and vascular calcification. Until adequately powered studies evaluating testosterone effects in hypogonadal men at increased CV risk are available (TRAVERSE trial), clinicians should ponder the use of testosterone in men with atherosclerotic cardiovascular disease and discuss benefit and harms with the patients.

## Introduction

Cardiovascular disease (CVD) is the leading cause of death worldwide, causing over 17 million premature deaths in 2016 [1]. The 2015 World Health Organization mortality registry has reported that CVD kills more women (55%) than men (45%), but the proportion of CV deaths before the age of 65 years is larger in men (30% compared to 26% in women) [2].

Atherosclerosis is a disease characterized by low-grade and chronic inflammation of the arterial wall which is triggered by subendothelial retention of plasma-derived apolipoprotein B (apoB)-containing lipoproteins in the inner layer of the arterial wall, the intima [3]. Besides this lipid-inflammatory etiology, advancing age is itself an established risk factor for CVD, being a component of all major lifetime risk estimate calculators [4, 5]. In view of the overall increase in life expectancy, evidence-based strategies to prevent CVD are needed in older people. This has drawn attention to the role of sex steroids, particularly testosterone, in cardiovascular health [6]. Declining testosterone levels in older men have been associated with the process of aging called “late-onset hypogonadism” (LOH), [7, 8] a clinical syndrome (usually associated with overweight/obesity requiring a specific rehabilitation) which results from failure of the testes to produce physiological concentrations of testosterone and/or a normal number of spermatozoa due to abnormalities at one or more sites of the hypothalamic–pituitary–testicular axis (HPT).

Testosterone deficiency has been generally linked to a rise in major adverse cardiovascular events (MACEs), especially myocardial infarction (MI) and stroke. However, whether testosterone replacement therapy is beneficial is still debated [9–11]. In 2010 the Food and Drug Administration (FDA) raised concerns on the potentially increased risk of CVD events upon testosterone administration after the premature interruption of the Testosterone in Older Men With Mobility Limitations (TOM) study [12]. Thus, in March 2015, all US commercial testosterone products underwent an FDA-mandated label change that restricted the prescription of these medications to men with hypogonadism of known etiology and included a warning about the risk of heart attacks and strokes [13].

Testosterone treatment has been associated with increased wellbeing and improved CV symptoms (angina, claudication). An extensive overview by Oskul et al [14] documented the benefit of testosterone in angina, with raised mean time to 1-mm ST-segment depression on exercise stress testing from 309 seconds at baseline to 343 seconds after 4 weeks and 361 seconds after a 12-week treatment [15]. Intracoronary testosterone (10^−10^ to 10^−7^ mol/L) in non-hypogonadal men induced a coronary vasodilatation of up to 4.5% compared to baseline [16]. A similar positive vasodilator activity was reported on brachial arteries following oral testosterone administration [17]. A number of potential mechanisms have been hypothesized, but there is clear evidence that the vasodilator activity is not linked to stimulated nitric oxide [18]. However, in peculiar conditions, as in the case of trans men undergoing hormone-affirming replacement therapy, exposure to testosterone may worsen endothelial function [19].

Finally, it should be considered that in men with coronary heart disease, testosterone deficiency seemed to be a relevant medical condition. As reported in a longitudinal study with a follow-up of 6.9±2.1 years, in men with vascular disease, testosterone deficiency was associated with premature death. Levels < 15.1 nmol/L corresponded to a HR of 1.86 (95%CI 1.1-3.2) for all-cause mortality and of 2.50 (95%CI 1.2-5.3) for vascular mortality [20].

Considering that the CV benefit and safety of treating low testosterone conditions have not been definitely proven and controversies still persist, this review article focuses on these controversies and on the potential risks and benefits of testosterone supplementation on CV health.

Background reasons for the so far inconclusive results among studies are (i) differences in dose regimens, (ii) insufficient statistical power for clinical events, or (iii) inclusion criteria allowing the recruitment of healthy asymptomatic men with low or low-normal testosterone. The effects of testosterone on atherosclerosis will be also reviewed in this context, including impact on platelet aggregation [21], high-density lipoproteins (HDL) functionality and serum capacity to load macrophage with cholesterol [22].

To pursue this aim, by using pubmed.gov, the following algorithm was used: *hypogonadism* or *testosterone replacement therapy* or *testosterone deficiency* AND *atherosclerosis* AND *cholesterol efflux capacity* AND *cholesterol loading capacity* AND *coagulation* AND *inflammation-related atherosclerosis* AND *lipoproteins* AND *major adverse cardiovascular events* AND *QT interval* AND *subclinical atherosclerosis* AND *thromboembolism*. Relative to clinical studies, the search for literature comprised observational, retrospective, interventional and prospective studies. BG and MR screened titles and full text of papers identified in our search. In the present review, testosterone levels have been reported in different units, as in the original manuscripts. 1nmol/L testosterone is equal to 28.842 ng/dL.

## Male hypogonadism: definition and classification

Male hypogonadism historically classified according to either the site of origin (primary/peripheral, secondary/central) or to the gonadotropin levels (hypo- or hyper-gonadotropic) is a clinical syndrome associated with androgen deficiency that originates from a putative damage to the hypothalamus-pituitary-testis (HPT) axis [23, 24]. As summarized in Table 1, primary hypogonadism is typically hypergonadotropic, being related to specific testicular damage resulting from different causes, either congenital or acquired. Conversely, secondary or central hypogonadism are usually hypogonadotropic and caused by congenital or acquired hypothalamic-pituitary damage. Both forms of hypogonadism are indistinctly characterized by low total testosterone, although so far there is no internationally recognized threshold to refer to. Usually, a testosterone value below 8 nmol/L is considered pathological, whereas a value above 12 nmol/L is unlikely indicative of hypogonadism [25–29]. Nevertheless, there is a gray area regarding testosterone between 8 and 12 nmol/L that needs to be carefully considered, as should the calculation of free testosterone, a parameter rated as normal when above 225 pmol/L [25–29]. The clinical phenotype is strictly related to the onset of the androgenic defect (Table 2). Forms associated with a very early onset (some congenital and genetically determined), also during fetal life, affect the neonatal masculine phenotype and lead to specific signs, such as cryptorchidism (especially bilateral), microphallus and/or hypospadias. Other forms become evident in the peripubertal period and are characterized by absent or incomplete pubertal development. Finally, forms that become manifest only in the adulthood – LOH – are characterized by specific signs and symptoms (hypoactive sexual desire, impaired spontaneous nocturnal/morning erections and/or sexual-related erections) and less specific signs and symptoms (loss of muscle mass, increased body fat, anemia, osteoporosis, depressed mood, decreased vitality, sweating, and hot flushes) [30, 7]. In adult men older than 40 years, a biochemical finding of secondary or primary hypogonadism is frequent, being present in around 12% and 2%, respectively, of men of the European general population. According to the majority of Andrology Societies [25–29], and to the Endocrine Society [10], only men with testosterone deficiency that present with specific symptoms can be categorized and treated for true hypogonadism [31]. Forms of late-onset hypogonadotropic hypogonadism may have a genetic predisposition presenting an enrichment of rare variants in genes typically associated with congenital hypogonadotropic hypogonadisms [32]. This is more likely, considering subjects with total testosterone levels below 6 nmol/L and with an onset before 41 years of age [32]. Moreover, adult-onset hypogonadotropic hypogonadism has been associated with minor pubertal delay, thus, suggesting an underlying pre-existing mild impairment of the hypothalamic–pituitary–gonadal (HPG) axis [33–35]. The adult-onset hypogonadotropic hypogonadism phenotype shows non-reproductive clinical manifestations and may share features related to the classical pre-pubertal congenital hypogonadotropic hypogonadism [36], further supporting the hypothesis of a shared origin.

**Table 1.** Causes of male hypogonadism

Hypogonadism	Congenital	Acquired
Primary	- Chromosomal abnormalities (*e.g.*, Klinefelter syndrome, De La Chapelle syndrome) - Genetic causes (*e.g.*, Y chromosome microdeletions, androgen insensitivity, enzymatic defects in androgens biosynthesis, myotonic dystrophy, LHR/FSHR resistance) - Congenital anorchia - Cryptorchidism - Sertoli cell only syndrome - Varicocele - Idiopathic	- Viral orchitis (*i.e.*, mumps) - Autoimmune - Testicular trauma - Testicular torsion - Iatrogenic (*i.e.*, radio- or chemotherapy, drugs, orchiectomy) - Secondary varicocele
Secondary	- Kallmann syndrome - Congenital hypogonadotropic *Hypogonadism (CHH)* - Isolated LH or FSH deficiency - Multiple Pituitary Hormone *Deficiency (MPHDs)* - Prader-Willi syndrome - Laurence-Moon-Biedl syndrome	- Severe chronic illness (*e.g.*, renal insufficiency, hepatic insufficiency) - Hyperprolactinemia - Excessive exercise - Nutritional deficiency and starvation - Obesity/metabolic syndrome/diabetes mellitus - Acquired MPHDs (*e.g.*, hypothalamic-pituitary lesions, traumatic brain injuries, vascular brain accidents, hypophysitis, hemochromatosis, sarcoidosis) - Drugs (*e.g.*, gonadal steroids, glucocorticoids, opiates, GnRH analogues) - Iatrogenic (*e.g.*, pituitary surgery, radiotherapy) - Idiopathic
Combined	Late Onset Hypogonadism (LOH)	

*CHH*, Congenital hypogonadotropic hypogonadism; *FSH*, Follicle Stimulating Hormone; *FSHR*, Follicle Stimulating Hormone Receptor; *GnRH*, Gonadotropin-releasing hormone; *LH*, Luteinizing Hormone; *LHR*, Luteinizing Hormone Receptor; *MPHD*, Multiple pituitary hormone deficiency

**Table 2.** Signs and symptoms of male hypogonadism according to the onset of the disease

Age of Onset	Signs & Symptoms
Fetal/Neonatal	Microphallus
	Hypospadias
	Cryptorchidism
Peri-pubertal	Absent or incomplete puberty
	Eunuchoid proportions
	Underdeveloped genitalia
Adult	Erectile dysfunction (spontaneous and/or sex-related)
	Hypoactive desire
	Infertility
	Osteoporosis
	Depression/Decreased vitality
	Asthenia
	Increased body fat/decreased lean mass
	Hot flushes/sweating
	Anaemia

Finally, although not in the remit of the present review article, it is worth mentioning that the treatment of male hypogonadism will be tailored to patients to be treated, taking into account age, specific forms of hypogonadism and any eventual associated comorbidities [37, 38, 10, 29]. Adult-onset forms of male hypogonadotropic hypogonadism can benefit from a dual therapeutic choice that includes the use of exogenous gonadotropins to stimulate not only testicular function but also to allow spermatogenesis and fertility of the patients. The primary forms, characterized by damage of the testis, can only be treated with testosterone replacement therapy. To date, there are several pharmaceutical forms of testosterone approved by the main international drug Agencies, including the Food and Drug Administration and the European Medicines Agency. Testosterone pharmaceutical formulations are usually classified by the route of delivery and can be divided in oral, buccal, nasal, subdermal, transdermal and intramuscular preparations. Specific advantages and disadvantages of each formulation has to be considered in the therapeutic decision process after a patient-centered approach that explicit potential risks and benefits [39].

## Impact of testosterone in atherosclerosis

### Subclinical atherosclerosis

Carotid intima-media thickness (cIMT), a biomarker of subclinical atherosclerosis positively associated with the risk of CVD events [40, 41], may be influenced by sex hormones. Although not reporting testosterone levels, prominent in this area were the observations by O’Leary who reported that in older adults (72.5±5.5 years) without a history of CVD, rises in cIMT were directly associated with an increased risk of myocardial infarction and stroke, people in the highest quintile having a 3.9 times higher risk as those in the lowest quintile [42]. This has been confirmed over the years in several cross-sectional studies reporting that in men with symptoms and hormonal levels identifying LOH, IMT of the carotid artery was inversely associated with total testosterone levels [43, 44] as was when free testosterone was considered (β: -3.57; 95% CI, -6.34 to -0.80) [45].

An important role in this association is played by inflammation. In older people, with high inflammation (high-sensitivity C-reactive protein (hsCRP) ≥ 2 mg/L), mean cIMT was significantly higher when testosterone levels were ≤ 3.2 ng/mL [46]. An opposite conclusion came from the results of the randomized controlled trial (RCT) Testosterone's Effects on Atherosclerosis Progression in Aging Men (TEAAM). The primary endpoint was to evaluate the effect of testosterone administration on the progression of subclinical atherosclerosis in 306 older men (67.6 years) with either low or low-normal testosterone (100–400 ng/dL or free testosterone < 50 pg/mL). Three years’ administration of testosterone (7.5 g of 1% testosterone) led to a nonsignificant rate of change in cIMT: 0.010 mm/year in the placebo group vs 0.012 mm/year in the testosterone group. In addition, coronary artery calcium scores did not differ between groups (the mean difference was -10.8 Agatston units/year; 95%CI -45.7 to 24.2; p=0.54), highlighting the neutral effect of testosterone supplementation compared to placebo in the progression of calcified plaques [47] (Table 3). However, some men with a history of MI or stroke were not enrolled, while those on phosphodiesterase type 5 inhibitors were assigned to the control group [48].

**Table 3.** Impact of testosterone in atherosclerosis

Study	Population’s characteristics	Outcomes
TEAAM [47]	306 men (67.6 years) with testosterone levels of 100-400 ng/dL and free testosterone < 50 ng/dL. 7.5 g of 1% testosterone were given for 3 years	- The rate of changes in intima-media thickness was 0.010 mm/year in the placebo group and 0.012 mm/year in the testosterone group. - The rate of changes in coronary artery calcium was 41.4 Agatston units/year in the placebo group and 31.4 Agatston units/year in the testosterone group.
TTrials [51]	170 men (65 year or older) with testosterone levels < 275 ng/dL. Testosterone 1% gel in a pump bottle was given for 12 months	- noncalcified plaque volume raised from 204 mm^3^ to 232 mm^3^ in testosterone group and from 317 mm^3^ to 325 mm^3^ in the placebo group. - total plaque volume increased from 272 mm^3^ to 318 mm^3^ in the testosterone group and from 499 mm^3^ to 541 mm^3^ in the placebo group. - CAC score dropped from 255 to 244 Agatston units in the testosterone group and raised from 494 to 503 Agatston units in the placebo group.
Offspring and Third Generation cohorts of the Framingham Heart Study [54]	1654 community-dwelling men. Testosterone was 616 ng/dL and free testosterone was 111 pg/mL.	- CAC decreased by -23% every 100-ng/dL between-subjects increase in testosterone.
Athero-Express Biobank Study [55]	611 specimens. Testosterone was 12.3 nmol/L and E2 was 92.8 pmol/L	- The testosterone/E2 ratio was negatively associated with plaque calcification: OR 0.816, 95%CI: 0.666 - 0.924. - In the low testosterone/E2 ratio group, HR for MACE was 1.67 (95%CI: 1.02–2.76). In the group with BMI ≥ 25 kg/m^2^ the HR was 2.42 (95% CI: 1.09–5.38).
Idiopatic or genetic (Kalmann and Klinefelter syndromes) forms of hypogonadism [22]	20 patients. Testosterone was 4.21 nmol/L	- Decrement of total HDL CEC (-16.2%). - Rise in serum CLC (+43%).
Database of a commercial clinical laboratory [69]	10,041 men (58 years). Testosterone was 420 ng/dL	- 1,518 men with testosterone levels < 250 ng/dL (vs those with testosterone levels > 250 ng/dL) had significant elevated levels of hsCRP, IL-6, IL-17A, and TNF-α.
Randomized, single-blind, placebo-controlled, crossover study [74]	Testosterone replacement (Sustanon 100) vs placebo (62 ± 9 years). Total testosterone was 4.4 ± 1.2 nmol/L	- Compared to placebo, testosterone reduced TNFα (−3.1 ± 8.3 pg/mL; p=0.01), IL-1β (−0.14 ± 0.32; p=0.08) and increased IL-10 (0.33 ± 1.8; p= 0.01).

CAC, coronary artery calcification; CEC, cholesterol efflux capacity; c-IMT, carotid intima-media thickness; CLC, cholesterol loading capacity; E2, estradiol; hsCRP, high-sensitivity C-reactive protein; HR, hazard ratio; IL, interleukin; OR, odd ratio; TEAAM, Testosterone’s Effects on Atherosclerosis Progression in Aging Men; TTrials, Testosterone Trials; TNF-α, tumor necrosis factor; 1nmol/L testosterone is equal to 28.842 ng/dL

The evaluation of plaque volume in coronary arteries by computed tomographic angiography was one of the goals of the cardiovascular sub-trial of the Testosterone Trials (TTrials) enrolling men 65 year or older with testosterone levels < 250-275 ng/dL on two occasions and self-described symptoms of “male hypogonadism” [49], the noncalcified plaque volume was estimated by the means of low-attenuation plaque, fibrous-fatty plaque, and fibrous plaque; total plaque volume was defined as noncalcified plaque plus dense calcium plaque. Specifically, the TTrials were a set of seven trials aimed at evaluating whether transdermal testosterone treatment of hypogonadal elderly men with low serum testosterone concentrations accompanied by symptoms and objective evidence of impaired mobility and/or reduced libido was effective in improving mobility (Physical Function Trial), sexual function (Sexual Function Trial), fatigue (Vitality Trial), cognitive function (Cognitive Function Trial), hemoglobin (Anemia Trial), bone density (Bone Trial), and coronary artery plaque volume (Cardiovascular Trial) [49]. This last was the last trial to be included and was underpowered to answer the question whether testosterone would increase, decrease or have no effect on cardiovascular events in hypogonadal men. The initial dosage of the testosterone gel was 5 mg with adjustments during the study to attain levels of testosterone in the range 500-800 ng/dL (reviewed in [50]). One year-treatment with testosterone (1% gel in a pump bottle) resulted in a significantly greater increase in noncalcified plaque volume, as assessed by computed tomographic angiography. From baseline, people assigned to testosterone had median values rising from 204 mm^3^ to 232 mm^3^, compared to the placebo group (from 317 mm^3^ to 325 mm^3^; between groups p= 0.003) [51]. The increment in noncalcified plaque volume was found in 70% of men given testosterone, compared to 54% to those in the placebo group, whereas the proportion of those showing a plaque regression was 27% in the testosterone group and 45% in the placebo group [52]. Relative to the secondary endpoint of total plaque volume*,* this rose from 272 mm^3^ to 318 mm^3^ in the testosterone group and from 499 mm^3^ to 541 mm^3^ in the placebo group (between groups p= 0.006). Finally, the coronary artery calcification scores were essentially unchanged in both groups (Table 3) [51]. These findings were the object of criticism since it cannot be assumed that a rise in plaque volume will always lead to a limitation of vascular lumen, since vascular remodeling may lead to a maintenance of luminal volume [53].

Vascular calcification was also the object of a cross-sectional analysis of data from men in the Offspring Cohort and the Third Generation Cohort of the Framingham Heart Study [54]. Vascular calcification at all sites was negatively associated with testosterone and calculated free testosterone. This trend was attenuated and became statistically nonsignificant after adjustment for traditional CV risk factors (Table 3).

A possible shortcoming in the relationship between sex hormones and CVD risk is that most studies did not look at the ratio between testosterone and estradiol (E2). Yet, it has not been defined whether an interplay, a codependency or rather a synergist effect between these hormones does exist. In a trial that involved the analysis of 611 specimens from male patients who underwent carotid endarterectomy in the Athero-Express Biobank Study, the testosterone/E2 ratio was negatively associated with plaque calcification (Odds Ratio (OR): 0.816; 95%CI: 0.666 - 0.924; p= 0.044), total number of plaque neutrophils (p= 0.012)), and interleukin-6 (IL-6) (p= 0.009). An unfavorable inflammatory pattern was found in patients with low testosterone/E2 ratio, *i.e.*, elevated hsCRP levels: 2.81 μg/mL *vs* 1.22 μg/mL in those with high testosterone/E2 ratio. Finally, low testosterone/E2 ratio independently predicted future major cardiovascular events (hazard ratio (HR) 1.67; 95%CI: 1.02–2.76, an effect even stronger in obese men (HR 2.42; 95% CI: 1.09–5.38) (Table 3) [55].

### Lipoprotein functionality

The effects of testosterone replacement therapy on cholesterol levels have been investigated by several Authors with no clear conclusions. Testosterone may affect low-density lipoprotein cholesterol (LDL-C) by inhibiting 7α-hydroxylase, a key enzyme in bile formation and cholesterol removal [56]; it may reduce high-density lipoprotein cholesterol (HDL-C) levels by raising the activity of hepatic lipase [57] contrary to estrogens [58]. Testosterone is responsible for the hydrolysis of the triacylglycerol component of circulating chylomicrons and very low-density lipoproteins (VLDL) [59]. HDL represent a heterogeneous class of lipoproteins with several CV protective functions, the promotion of cholesterol efflux from arterial macrophages of arterial having been most extensively described.

The quantitation of HDL cholesterol efflux capacity (CEC), has been found to be inversely related with incident CV events, thus leading to the hypothesis that HDL function is a better predictor of CVD risk compared to absolute plasma HDL-C level [60]. When evaluating the relationship between testosterone levels and CV risk associated to changes in lipoprotein functions, Rubinow et al. did not find any differences in HDL CEC after testosterone replacement in older hypogonadal men; conversely, testosterone was associated to an altered HDL proteome, *i.e.,* raised paroxonase-1 and fibrinogen-α-chain and reduced apolipoprotein A-IV, apolipoprotein C-I and paraoxonase 3 [61]. When a gonadotropin-releasing hormone antagonist was administered to middle-aged males, the resulting hypogonadism increased HDL-C without affecting the effluxing capacity [62]. The same pharmacological castration procedure in young healthy men increased both HDL-C and HDL CEC [61]. We have recently demonstrated that in cases of genetic and idiopathic hypogonadisms, very low levels of testosterone were associated with an impaired HDL CEC. This finding was confirmed when the main cellular cholesterol efflux pathways (ATP-Binding Cassette transporter A1 and ATP-Binding Cassette transporter G1) were considered individually [22]. Other parameters worthy of consideration are the qualitative and/or compositional modifications of HDL particles, *i.e.*, reduction in HDL_2_ subtractions in hypogonadal men [63] or changes in the HDL proteomic cargo [61]. HDL CEC only reflects the movement of cholesterol out of cells, whereas the cellular lipid trafficking is bidirectional, being the balance of efflux and influx processes. While it is unquestionable that low density lipoprotein (LDL) is causative in CVD [64], the serum cholesterol loading capacity (CLC) in macrophages has been less extensively explored [65]. In the idiopatic or genetic (Kallmann and Klinefelter syndromes) forms of hypogonadism, CLC is raised independently of LDL plasma concentrations, clearly indicating that the likely rise of arterial wall foam cell formation may contribute to a higher CV risk (Table 3) [22].

### Inflammation

Beyond the well-known role of LDL lipids in in atheroma development [66], data from the proof-of-concept Canakinumab Anti-Inflammatory Thrombosis Outcome Study (CANTOS) [67] clearly identified inflammation as a key biological trigger of atherosclerosis. Epidemiological studies evaluating the link between endogenous testosterone and CVD inflammatory markers, mostly CRP and IL-6, reported either an inverse or a lack of association [68]. These discrepancies may rely on the following postulations: (i) the impact of age, *i.e.*, negative associations between testosterone levels and CRP having been reported in relatively younger patients, a lack of association being described in older population groups; (ii) role of the adipose tissue, as when adjusting data for waist circumference or when enrolling obese patients a lack of association was found [14]. Among 10,041 male patients with median age 58 years (range 18–97 years), 1,518 men with testosterone levels < 250 ng/dL had significantly elevated hsCRP, IL-6, IL-17A, and tumor necrosis factor (TNF)-α, when compared to men with testosterone levels ≥ 250 ng/dL [69]. Finally, a role has been attributed to the NACHT-, LRR- and pyrin domain-containing 3 (NLRP3) pathway [70], a general mediator of arterial tissue inflammation [71]. In C57Bl/6J mice, testosterone administration induced vascular dysfunction through the generation of mitochondrial reactive-oxygen species, an effect not observed in mice lacking NLRP3 [70].

Data from RCTs have generally shown that inflammatory biomarkers linked to CV risk (TTrial study) were not reduced to a larger extent after testosterone 1% gel. There were no differences from baseline in d-dimer (a marker of fibrinolysis) changes: +0.1 mg/L in both arms; CRP decreased by 0.7 mg/L with testosterone and 0.1 mg/L with placebo (difference between groups: -0.6 mg/L, p=0.11); IL-6 levels were raised by 0.9 pg/mL with testosterone and by 0.2 pg/mL with placebo (p= 0.67) [72].

The current interest in the inflammatory causes of acute CVD and the predictive value of circulating biomarkers, particularly hsCRP, has resulted in only limited interventional data on testosterone. In a large sample (n= 2,301) of ethnically diverse men aged between 30 and 79 years there was a clear inverse association between CRP and testosterone levels [73]. These findings confirm a prior study [74] in which testosterone replacement (100 mg testosterone esters) vs placebo over 3 months led to significant reductions of TNFα, and IL-1β with a rise of IL-10 (Table 3). Similar findings were reported by Corrales et al [75] in a small study on type 2 diabetic men with androgen deficiency, where treatment for up to one year with testosterone enanthate (150 mg every two weeks) led to a dramatic drop in the production of the cytokines IL-1β, TNF-α and IL-6 by isolated circulating antigen-presenting cells. Further supporting data came from a study that demonstrated that long-term (24 months) testosterone undecanoate administration in patients with metabolic syndrome led to a decrement in the levels of hsCRP and cIMT [76].

The inverse paradigm, *i.e.*, the effect of inflammation on testosterone levels, was the object of an RCT involving 67 men with metabolic syndrome: 33 were given 100 mg of anakinra (a recombinant human IL-1β receptor antagonist) bid for 4 weeks vs placebo. This treatment significantly raised testosterone levels (1.2 nmol/L) vs placebo (0.96 nmol/L; 95%CI 0.29-1.89 nmol/L). When patients were stratified according to the baseline CRP threshold of 2 mg/L, the effect was more consistent in those with CRP > 2 mg/dL, achieving a significant between-group difference of 2.14 nmol/L (95%CI 0.11-4.17). This stepwise increment was more pronounced in patients who reached a reduction > 1 mg/L after one week, which translated into a rise of 2.8 nmol/L in testosterone levels. The human IL-1β receptor antagonist did not, however, rescue severity of symptoms related to hypogonadism, or associated fatigue [77].

### Coagulation and thromboembolism

Beyond inflammation and cholesterol accumulation, platelet hyperactivity plays a key role in the genesis and in progression of atherothrombosis, stimulating plaque progression and thrombus formation [78]. Confirmations on the hemostatic properties of testosterone came from a study in men (age 62.9 ± 1.7) with mean testosterone levels of 4.49 (4.41-4.71) ng/mL (15.6 (15.3-16.3) nM). In this study, testosterone and dihydrotestosterone were negatively associated with platelet aggregation in response to arachidonic acid or collagen. The testosterone-driven antiplatelet activity is not dose-dependent as it is exerted even at low testosterone concentrations, in the range of hypogonadism [21]. It has been inferred that testosterone inhibits platelet aggregation by stimulating endothelial nitric oxide synthase and vascular endothelial cell growth [79], and confirmation of this came from a study on patients affected by Klinefelter Syndrome. Stimulation of platelets with arachidonic acid (0.2 and 0.4 mM) induced an irreversible aggregation in 70% of Klinefelter Syndrome patients compared with 15% of controls. These findings were corroborated by the detection of raised levels of circulating 8-iso-prostaglandin F2α (8-iso-PGF2α) and 11-dehydro-thromboxane B2 (11-dehydro-TXB2), recognized markers of oxidative stress and platelet activation [80]. Conversely, an earlier RTC had detected a neutral effect of testosterone in a setting of 46 men with chronic stable angina. The administration of physiological doses of supplemental testosterone led to no differences in the levels of plasminogen activator inhibitor-1, fibrinogen, tissue plasminogen activator or hemoglobin concentrations [81].

However, the warning coming from the FDA relative to the association between testosterone therapy and the risk of thromboembolism should not be underestimated. Although there is no doubt on the erythrogenic effect of androgens [82] that leads to an increase in the levels of hemoglobin and hematocrit, the association between testosterone-induced erythrocytosis and subsequent risk of venous thromboembolism remains doubtful. No increment of venous thromboembolism was found in two meta-analyses (OR 1.9 (95%CI 0.75-5.17) [83] and OR 1.41 (95%CI 0.96-2.07) [84]), an evidence persisting regardless of the different routes of administration (transdermal or intramuscular) [85]. Conversely, different conclusions were reached by Martinez et al [86] who reported an increased risk of venous thromboembolism within six months of testosterone therapy initiation: a rate ratio of 1.63 (1.12 to 2.37) became 1.00 (0.68 to 1.47) after six months, and 0.68 (0.43 to 1.07) after treatment cessation [86]. A case-crossover study analyzing data on 39,622 men concluded that testosterone raises venous thromboembolism shortly after treatment initiation. The risk estimates were approximately doubled (OR 2.32, 95%CI 1.97-2.74) compared to men without hypogonadism, the risk being larger in the first 3 months after initiation. This evidence did not vary by route of exposure [87].

In conclusion, for populations at high CV-risk, it is advisable to check for thrombophilic conditions, antiphospholipid antibody syndrome and prothrombin gene mutations before initiating any form of testosterone treatment. Transdermal or subcutaneous formulations should be strongly considered in at-risk populations [88]. Overall, patients that require testosterone treatment must undergo a complete blood count not only at baseline but also after 3-4 and 12 months, and then annually [82]. Considering that the effect of testosterone on hematocrit is dose-dependent and influenced by age, during testosterone treatment hematocrit should be < 54% and dose adjustment or temporary treatment interruption has to be considered to keep hematocrit below 54% [89].

## Testosterone replacement therapy and cardiovascular outcomes

### Observational and retrospective studies

Observational studies suggest an association between low endogenous testosterone levels and the increased risk of CV or all-cause mortality [14, 90] (Table 4). The strongest evidence came from a meta-analysis including 12 observational studies comprising 16,184 subjects (mean age 61 years and median follow-up 9.7 years) of which 7 studies specifically related to CVD mortality (n= 11,831) [90]. The risk of low testosterone was especially high for men over 60 (Relative Risk (RR) 1.54, 95% CI 1.28–1.85 when comparing the highest to the lowest tertile). The major limitations of these findings were the absence of a common cut-off for the definition of low testosterone across studies leading to a considerable between-study heterogeneity, and the potential unadjusted confounders [6]. An inverse association between testosterone concentrations and CV mortality was found among 2314 men of the prospective EPIC-Norfolk (European Prospective Investigation into Cancer in Norfolk) study. In this nested-control study, every 6-nmol/L rise in serum testosterone corresponded to a 17% reduction of the risk (OR 0.83, 95% CI 0.74 to 0.94) [91]. However, the study reported neither levels of calculated nor available free testosterone, a parameter more accurate than total testosterone in obese or diabetic individuals [92]. Similar conclusions were drawn in the Rancho Bernardo Study with a follow-up of 11.8 years. Men with total testosterone levels < 241 ng/dL had a 38% higher risk of CVD (HR= 1.38, 95%CI 1.14–1.71) [93]. A further confirmation was reported in the larger cohort of MrOS (Osteoporotic Fractures in Men) study (n= 2416). High serum testosterone levels (680±127 ng/dL) predicted a reduced 5-year risk of CV events (HR of 0.77, 95%CI 0.60-0.98) [94]. This evidence was also reported in the case of ischemic stroke with extremely low levels of testosterone (173 ng/dL) associated to a HR of 1.34 (95%CI 1.05-1.72) [95].

**Table 4.** Association between low testosterone levels and total or cardiovascular mortality in population-based cohort studies

Years	Number of patients	Mean follow-up (yr)	Mean age (yr)	Results^a^ Effect size (95% CI)
Barrett-Connor (1988) [154]	872	12	63	RR 0.87 (0.61-2.08)
Smith (2005) [81]	2323	16.5	52.1	RR 1.39 (1.00-1.93)
Araujo (2007) [155]	1686	15.3	55	RR 0.81 (0.52-1.31)
Khaw (2007) [91]	2314	7	67.3	RR 1.82 (1.17-2.74)
Laughlin (2008) [93]	794	11.8	73.6	HR 1.38 (1.02-1.85)
Haring (2010) [156]	1954	7.2	58.7	RR 4.93 (1.21-20.26)
Menke (2010) [157]	1114	8	40	RR 1.24 (0.85-1.78)
Vikan (2010) [158]	1568	11.2	59.6	RR 1.09 (0.74-1.63)
Haring (2012) [156]	2039	5.5	20-79	RR 2.05 (1.61-2.60)^b^
Pye (2014) [159]	2599	4.3	40-79	RR 2.30 (1.20-4.20)^c^
Shores (2014) [100]	1032	9	76	RR 1.03 (0.95-1.11)^d^

^a^Relative risk comparing the upper versus the lower tertile of testosterone levels.

^b^Relative risk comparing the lowest 10^th^ percentile versus the others.

^c^Relative risk comparing patients with low testosterone levels (< 8 nM) vs eugonodal.

^d^Per standard deviation decrease.

Abbreviations: CI, confidence intervals; HR, hazard ratio; yr, year; RR, relative risk

Longitudinal studies have also confirmed that symptoms and signs related to hypogonadism are all raised in patients with a history of CVD, although in those at higher CV risk, hypogonadism (total testosterone < 350 ng/dL – 12 nM) was associated to a lower incidence of new CV events [96]. It is likely that endogenous testosterone level is a marker of general health, and those who are at higher risk of mortality have lower testosterone concentrations.

In a large population of men with acute coronary syndrome, we have investigated the prevalence of low total endogenous testosterone levels (< 300 ng/dL or 10.4 nM) and found it close to 40% [97]. Observational studies can only show associations but cannot infer about direct causality or differentiate with reverse causality for medical recommendations.

In practice, data from pharmacy claims indicate that testosterone replacement prescriptions have increased over time in elderly men [98]. Among men with androgen deficiency, dispensed testosterone prescriptions are associated with a lower risk of CV outcomes, *i.e.*, acute myocardial infarction, coronary revascularization, unstable angina, stroke, transient ischemia attack, and sudden cardiac death. Over a median follow-up of 3.2 years, testosterone replacement therapy led to a 33% reduction in the primary outcome (adjusted HR= 0.67; 95%CI 0.62-0.73) [99]. In an observational cohort study of 398 men with androgen deficiency (Northwest Veterans Affairs medical centers), the use of testosterone replacement reduced CV events by 39% compared to untreated subjects over a follow-up of 3.4 years [100]. A matched-case control analysis, involving 6,355 testosterone treated individuals vs 19,065 free from testosterone therapy individuals, showed no increased risk of MI (HR= 0.84, 95% CI 0.69–1.02), but even a potential decrease in a subset of patients with a high-risk profile [101]. Further evidence on this topic has come from a large retrospective study involving 1,470 patients with low testosterone levels and an history of MI. Testosterone replacement therapy did not increase the recurrence of MI but did reduce all-cause mortality only when testosterone levels were normalized [102]. Interestingly, Etminan et al in a large observational study found no association either between MI and past or current use of testosterone replacement therapy, although an increased risk was described in subjects at first use of testosterone replacement therapy [103].

Retrospective evaluation of 83,010 veterans with documented low testosterone levels showed that prolonged therapy with optimally dosed testosterone appears to be beneficial in terms of CV endpoints. With a mean age of 66 years, men were categorized into those treated with testosterone with normalization (gp1), testosterone treated without normalization (gp2) and not on testosterone [104]. With a follow up ranging between 4.6 and 6.2 years, the comparison between the gp1 and gp3 showed a reduction in the risk of all-cause mortality (HR= 0.44, 95%CI 0.42-0.46), MI (HR= 0.76, 95%CI 0.63-0.93) and stroke (HR= 0.64, 95%CI 0.43-0.96). Comparison between gp1 and gp2 resulted in a HR of 0.53 (95% CI 0.50-0.55) relative to all-cause mortality, of 0.82 (95%CI 0.71-0.95) relative to the risk of MI, and of 0.70 (95%CI 0.51-0.96) relative to stroke (HR: 0.70, CI 0.51-0.96) [104].

Opposite conclusions were reported in two retrospective studies [9, 105] that have, however, been widely criticized [106]. In the first trial by Veing et al, 8,709 men in the Veterans Healthcare System with low testosterone levels (<300 ng/dL or 10.4 nM) who underwent coronary angiography, the use of testosterone significantly raised adverse outcomes (all-cause mortality or hospitalizations for MI or ischemic stroke) over 3 years, compared with men without testosterone replacement [9]. A later official correction reported a 50% lower rate of CVD events in men on testosterone (10.1% *vs* 21.2%, respectively) [107] [108] This trial was, however, largely criticized as the values of testosterone levels achieved after treatment, *i.e.*, 332.2 ng/dL, lay just within the lower end of the normal range, thus indicating that many men were undertreated. Testosterone patches supplied 2.5 mg/24 h, whereas at that time the usual recommended dose was 5 mg. Another limitation was that only 60% of the treatment group had their testosterone levels retested during follow-up, with no knowledge as to whether one prescription meant 30 days or 90 days of treatment [109–111].

Finkle et al. provided similar negative evidence on CV endpoints following testosterone prescription. By using a large health-care database, cardiac risk was investigated in the 90 days post-testosterone prescription in 55,593 subjects, compared to a larger cohort (167,279) of similar patients receiving phosphodiesterase type 5 inhibitors. The overall rise in coronary risk was 36%, relative risk rising from 0.95 in subjects < 55 years to 3.43 in those aged > 75 years. The excess CV risk in patients below 65 years was restricted to those with pre-existing coronary conditions [105]. One of the major flaws of this study was the absence of a proper control group. The use of phosphodiesterase type 5 inhibitors in heart failure patients has in fact shown to improve left ventricular ejection fraction, diastolic function, exercise tolerance, and overall clinical conditions [112].

In line with these two reports, a population-based cohort study found that the use of testosterone associates with an increased risk of cerebrovascular and cardiovascular diseases, *i.e.,* the HR for the composite outcome was 1.21; 95% CI, 1.00-1.46. The risk was higher in the first 2 years of testosterone use. A second finding was a reverse causality, *i.e.*, a strong protective effect on mortality with current use. Patients who stopped testosterone replacement therapy a few weeks prior to cohort entry had a nearly twofold increased risk of dying (adjusted HR 1.72, 95%CI 1.21-2.45) [113, 114]. However, criticism was expressed regarding the use of a comparative cohort given 5α-reductase inhibitors, or the knowledge that testosterone deficiency itself is a risk for increased CV events. Indeed, some Authors claimed that data on MACE during the first 12 months could have be linked to hypogonadism rather than to testosterone treatment [115].

Finally, considering that hypogonadism is a comorbidity often present (25-40%) in men presenting with type 2 diabetes, an evidence confirmed by genetic studies [116], it is worth mentioning that the influence of baseline testosterone level on type 2 diabetes outcomes were evaluated in a 14-year follow-up study. During follow-up, the mortality rate was higher in patients with lower total testosterone (7.6±2 nmol/L) compared with normal baseline total testosterone (16.1±5 nmol/L), resulting in 36.1% of patients with normal baseline testosterone levels dying compared to 55.8% in those presenting with hypogonadism [117]. Similar conclusions were reached by human genetic studies that revealed that testosterone does play a causal role in the development of certain aspects of metabolic health (such as fasting levels of glucose and body fat levels). In men, each 1 SD increase in testosterone levels corresponded to an OR of 0.86 (95%CI: 0.76-0.98) to develop type 2 diabetes [118, 119].

Overall, retrospective analyses of data using electronic medical records are generally inconclusive, with major limitations: no randomized allocation, no prospective adjudication of CV events, confounding by indication (baseline risk of the participants determines the allocation to treatment), heterogeneity of patient populations, and intervention durations or differences in doses.

### Interventional studies

A meta-analysis including 27 intervention trials investigating the effects of testosterone treatment on 180 CV-related events in 2,994 older men suggested an increased risk of events with the use of testosterone (OR 1.54, 95% CI 1.09 – 2.18) [120]. The associated risks for testosterone-treated patients were higher in studies not funded by the pharmaceutical industry (OR 2.06, 95% CI 1.34 – 3.17), compared with industry-funded trials (0.89, 95% CI 0.50–1.60). Mechanistically, some data suggest an impact of testosterone therapy on atherosclerosis. A randomized trial including 138 men 65 years of age or older investigated the effect of testosterone (N=73) *vs* placebo (N=65) on the changes in coronary artery plaque volume over a period of 12 months [51]. At 12 months, the testosterone group had a significantly larger increase in median noncalcified plaque volume [51].

However, definitive data from randomized interventional trials with testosterone replacement therapy in older men are scarce [68]. In the widely quoted study on the effects of testosterone therapy on muscle performance and physical function in older men with mobility limitations (The TOM Trial) [12], 209 men (mean age, 74 years) with serum testosterone <350 ng/dL (12 nM) received a large testosterone dose by gel (100 mg testosterone daily), leading to high circulating testosterone levels, *i.e.*, 1,000 ng/dL (34.7 nmol/L) [121]. At the time of the trial, The Endocrine Society was recommending a testosterone goal of 400 to 500 ng/dL (13.9 to 17.4 nmol/L) in such patients [122].The study was prematurely halted due to a rise in CV risk (acute coronary syndrome, MI, angioplasty, syncope, atrial fibrillation, peripheral edema, stroke, elevated blood pressure), with an OR of 5.8 (95% CI 2.0–16.8) [12]. Besides the reasons discussed by the Authors, the results obtained may be the consequence of a rise in plasma estrogen. In view of the conversion of testosterone to estrogens, very high plasma testosterone levels might have led to hyperestrogenemia, with a consequently raised risk of CV events or thrombosis [123]. Another hypothesis accounting for the increased CV risk is raised erythropoiesis driven by testosterone, [124] or a higher degree of physical activity among subjects receiving testosterone supplementation [125]. Short-term physical exertion can precipitate sudden death, stroke, and acute left ventricular dysfunction [126].

The efficacy and safety of testosterone replacement on CV outcomes in older men was also evaluated in a systematic review and meta-analysis of 39 randomized controlled trials and 10 observational studies on testosterone replacement compared with placebo and did not indicate any significant increase in MI, stroke, or global mortality [127]. Secondary analyses have also shown that the increased CV risk upon testosterone treatment is present when frail men or those with body mass index (BMI) < 30 kg/m^2^ are considered. Conversely, testosterone treatment seems to be protective in trials enrolling subjects with a mean baseline BMI >30 kg/m^2^ [128]. Overall, there is still no definitive evidence that supports the risk or benefit of testosterone replacement on CV risk in men with hypogonadism [11].

Similarly, no increase in adverse events was reported in the previously mentioned TEAAM trial [12]. In this study where the carotid IMT and coronary calcium score vascular end-points were evaluated, no difference was noted between the two groups either for laboratory parameters, sexual function or health-related quality of life.

In this complex scenario, another category that should receive attention is represented by patients with type 2 diabetes*,* at higher CV risk. Although epidemiological studies have demonstrated that lower serum testosterone not only is common in men with established *type 2 diabetes* but also predicts future diabetic risks and increased mortality, the effects of testosterone treatment on androgen deficiency-like clinical features are not conclusive [129]. A placebo- controlled study was carried out in 220 hypogonadal men with type 2 diabetes or the metabolic syndrome treated for 6 months (plus 6 months allowing medication changes) with testosterone gel 2% vs placebo [130]. A reduction of the homeostasis model assessment-estimated insulin resistance (HOMA-IR) was noted (-15.2%), together with moderate improvements of glycemia, triglyceridemia and LDL-C, thereby indicating a potentially beneficial effect on insulin resistance, with minimal side effects. Hackett et al [131] reported that testosterone replacement therapy significantly improved HbA1c, total cholesterol, and waist circumference in men with type 2 diabetes, whereas Kalinchenko et al [132] found beneficial changes in weight, BMI and insulin, but without any improvement in serum glucose or lipid profile.

### A special condition: heart failure

Special consideration should be given to the value of testosterone in the pathogenesis and progression of heart failure (HF). Approximately 25% of men with chronic HF presented with biochemical evidence of testosterone deficiency [133]. Data from the ARIC (Atherosclerosis Risk in Communities) prospective studies (19.2 years of follow-up) showed that low levels of endogenous testosterone were associated with the development of HF [134], a finding in line with studies of Mendelian randomization describing that genetically predicted testosterone was positively associated with the risk of incidence of HF [135]. Cardiac cachexia, a metabolic and functional syndrome with poor prognosis, is characterized by particularly low testosterone levels with an increased risk of HF progression and mortality. The association of testosterone deficiency with myocardial cachexia and progression of HF led to the rationale that correcting testosterone deficiency in HF might be beneficial [133]. In patients with HF, testosterone supplementation was associated with improved exercise function, as well as metabolic parameters such as fasting glucose, fasting insulin, and insulin resistance. No safety concerns were reported in any of the trials; however, data were limited due to small samples and short follow-up (typically 12 weeks) [136]. Conversely, one of the largest prospective studies of testosterone replacement therapy in men with HF demonstrated that over a maximum follow-up of 12 months a significant benefit in functional capacity was obtained by raising the serum levels of testosterone by about 40%. In 76 men with an ejection fraction of 32.5+11%, testosterone administration compared to placebo improved exercise capacity up to 15±11% from baseline. In the active group, total testosterone increased within the normal physiological range (7.5–30 nmol/L) [137].

Opposite conclusions were, however, reported in a updated meta-analysis showing that in FH patients, testosterone supplementation within a physiological dose-range does not improve exercise tolerance (shuttle walk test or the six-minute walk test), cardiac function, quality of life, or clinical outcome. Of note, a subgroup analysis highlighted that subjects who had reached testosterone levels ≥ 25 nmol/L benefitted the most in terms of improved exercise capacity, as evaluated by shuttle walk test or the six-minute walk test [138].

Although no studies reported an improvement of systolic cardiac function after testosterone treatment in patients with HF, it is well-known that symptoms of HF are dictated by several factors other than cardiac dysfunction, including peripheral skeletal muscle and insulin resistance, that are potentially improved with testosterone replacement [139].

### Perspectives

The Study to Evaluate the Effect of Testosterone Replacement Therapy on the Incidence of Major Adverse Cardiovascular Events and Efficacy Measures in Hypogonadal Men (TRAVERSE) will be the first study on testosterone therapy adequately powered to assess CV events. Initiated in 2018, enrollment is planned to end in 2022 (NCT03518034). Briefly, this is a blinded and placebo-controlled study of topical testosterone replacement therapy in symptomatic hypogonadal men with an increased risk of CVD. Six thousand men aged 45 to 80 years with low serum testosterone (< 300 ng/dL or 10.4 nM) and at least one sign or symptom of hypogonadism and with evidence of CVD or at increased CVD risk will be randomized for a total duration of 60 months. The primary endpoint will be the first occurrence of a component of the composite of nonfatal MI, nonfatal stroke or death due to CV causes. The secondary outcomes include cardiac revascularization procedures (percutaneous coronary intervention and coronary artery bypass graft), incidence of prostate cancer, reduced sexual activity, depression, bone fracture, anemia and progression to diabetes [140].

Amid continued uncertainties [141], the aspects to be considered in future trials to succeed could be (1) the safety of different dosage forms (testosterone injections seemed to be associated with a greater risk of CV events, hospitalization, and deaths compared with topical formulation [142]), (2) the numerosity between treatment groups (at least 17,664 participants in each trial group), and (3) a duration approaching a decade [143].

## Conclusion

Testosterone replacement therapy is commonly prescribed to men and it is therefore essential to gain more reliable safety data for CV outcomes from large and long-term studies. Testosterone replacement therapy is recommended only in symptomatic men with hypogonadism and consistently low serum total testosterone [10]. Nonetheless, the FDA mandated pharmaceutical companies to add labeling information about a possible increased risk of CV events, while the European Medicine Agency concluded that there is no consistent evidence of an increased CV risk associated with testosterone therapy in hypogonadal men [11]. While it appears that testosterone replacement therapy does not cause a marked increase in the risk of CV events, another meta-analysis clearly highlighted that none of the studies that evaluated to estimate the risk (15 pharmaco-epidemiological and 93 RCT) had an enough long duration of exposure or were powered to exclude such a risk [128]. The 2020 clinical practice guideline by the American College of Physicians reached the same conclusion, *i.e.*, most of the studies on testosterone replacement were not designed to assess CV risk and thus excluded men with advanced heart failure or a recent history of myocardial infarction or stroke [144].

**Fig. 1.**
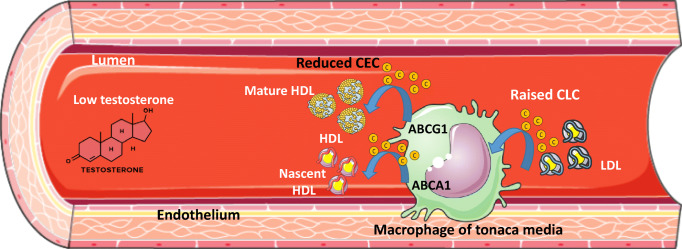
Effect of low testosterone levels on serum lipoprotein functions. Low circulating testosterone associates with a reduced total HDL CEC from macrophages of the arterial wall by negatively modulating the ABCA1- and ABCG1-mediated efflux pathways, and with a raised serum CLC. ABCA1, ATP-Binding Cassette transporter A1; ABCG1, ATP-Binding Cassette transporter G1; CEC, cholesterol efflux capacity; CLC, cholesterol loading capacity; HDL, high-density lipoprotein; LDL, low-density lipoprotein

On this matter, evaluating the lipid profile (*e.g.*, HDL-C levels) would *per se* not be enough, rather, it is the functional capacity of lipoproteins that would need to be considered. Since lipid trafficking is a balance between cholesterol efflux and influx, in hypogonadal men proatherogenic lipoprotein-associated changes have been associated with lower cholesterol efflux and increased influx, thus offering an explanation for a potentially increased CV risk (Fig. 1) [22]. Testosterone has also been described as a risk factor for venous thromboembolism due to a rise in (i) hematocrit and blood viscosity, (ii) platelet aggregation, and (iii) thromboxane A2 concentrations in platelets. Whether or not this is a direct effect driven by testosterone supplementation or due to a rise in the levels of estrogens remains unclear [83].

Among other aspects that are worth of consideration for testosterone treatment, there are the changes in epicardial fat thickness. A rise was detected in subjects with hypogonadal Klinefelter Syndrome, similar to that found in obese age-matched euploid subjects [145]. The accumulation of epicardial fat represents, along with visceral fat, a major contributor to CVD risk, above and beyond BMI [146], possibly due to localized release of inflammatory adipokines [147].

Finally, it is worth mentioning that testosterone use is associated also with regulation of ventricular repolarization by shortening the length of the QTc interval. A prolonged heart-rate QT is an independent predictor for cardiac, all-cause mortality [148] and *torsades de points* ventricular tachycardia [149]. Whether a high number of prolonged QT interval measurements was observed in hypogonadal men [150], the results from the TEAAM trials showed that testosterone replacement attenuates the age-related increase in QT interval duration [151]. Similar conclusions were reached in men aged ≥ 65 in whom the transdermal administration of testosterone attenuates drug-induced QT lengthening [152]. However, although the precise mechanism linking testosterone and QT interval is poorly understood, the stimulation of endogenous human *ether-a-go-go-related gene* potassium channels seems one of the most reliable hypotheses [153].

Overall, clinicians must exercise prudence in the use of testosterone in men with prevalent atherosclerotic coronary and cerebrovascular disease. Testosterone replacement in symptomatic elderly men with low testosterone levels should benefit from an individualized approach where uncertainties, risks and benefits of treatment can first be discussed with the patient.

## Data Availability

Not applicable

## Abbreviations

11-dehydro-TXB2: 11-dehydro-thromboxane B2
8-iso-PGF2 α: 8-iso-prostaglandin F2α
Apo: Apolipoprotein
BMI: Body Mass Index
CEC: Cholesterol Efflux Capacity
CHD: Cardiovascular Heart Disease
cIMT: Carotid Intima-Media Thickness
CLC: Cholesterol Loading Capacity
CV: Cardiovascular
CVD: Cardiovascular Disease
E2: Estradiol
FDA: Food and Drug Administration
HDL: High-Density Lipoprotein
HDL-C: High-Density Lipoprotein-Cholesterol
HF: Heart Failure
HOMA-IR: Homeostasis Model Assessment-Estimated Insulin Resistance
HPG: hypothalamic–pituitary–gonadal
HPT: Hypothalamus-Pituitary-Testis
HR: Hazard Ratio
hsCRP: High-sensitivity C-Reactive Protein
IL: Interleukin
LDL: Low-Density Lipoprotein
LDL-C: Low-Density Lipoprotein-Cholesterol
LOH: Late-Onset Hypogonadism
LRR: Leucine-Rich Repeat
MACEs: Major Adverse Cardiovascular Events
MI: Myocardial Infarction
NLRP3: NACHT-, LRR- and pyrin domain-containing protein 3
OR: Odds Ratio
RCT: Randomized Controlled Trial
RR: Relative Risk
TNF: Tumor Necrosis Factor
VLDL: Very Low-Density Lipoprotein

## Clinical Trial Acronyms

ARIC: Atherosclerosis Risk in Communities
CANTOS: Canakinumab Anti-Inflammatory Thrombosis Outcome Study
MrOS: Osteoporotic Fractures in Men
TEAAM: Testosterone Effects on Atherosclerosis in Aging Men
TOM: Testosterone in Older Men With Mobility Limitations
TRAVERSE: Testosterone Replacement Therapy on the Incidence of Major Adverse Cardiovascular Events and Efficacy Measures in Hypogonadal Men
TTrials: Testosterone Trials
